# Hearing and Examinations for the Public Service

**Published:** 1887-06

**Authors:** W. Laidlaw Purves

**Affiliations:** Aural Surgeon to Guy’s Hospital


### HEARING AND THE EXAMINATIONS FOR THE PUBLIC SERVICE.

*By* W. LAIDLAW PURVES, M.D., *Aural Surgeon to Guy's Hospital.*

A physical defect which does not seem to have received the attention it deserves in the examination of candidates for the different services is that of a deficiency in the normal hearing power. In the case of the eye, any marked imperfection of vision in both eyes generally forces itself upon the observer by the peculiar gait, or position of the head, or closure of the lids, which the sufferer employs to give him his best possible unaided vision. But a loss of hearing power much below the normal, either in both ears or limited to one ear, may escape detection by a careful observer who does not have recourse to more minute methods of investigation for determining the acuteness of hearing. This arises partly from the fact that ordinary conversation is carried on in a much louder tone than is necessary for the proper hearing thereof. With two normal ears the same conversation could be carried on with half the volume of sound, or even in a whisper; and, as the pica or long primer types are employed by the popular publisher to give comfortable and easy reading to eyes much under the normal in acuteness, so does the barrister or lecturer gauge, as far as he can, an intensity of sound which will give his audience easy hearing without straining. But, in the case of the candidate for admission to any of the services, not merely is it necessary for him to have the vision and hearing which can carry him through the daily avocations of private life, but he ought to have a standard normal acuteness of both organs on both sides. With only one sound and normally acute eye or ear, a man may pass through life without anyone, perhaps even himself, having discovered that he was partially blind or deaf; but he is essentially a one-barrelled man, and his chances of remaining with good vision and hearing during life are about 50 per centum less than those of a man with two normally acute eyes and ears.

Attention has been lately called to a standard of vision which is necessary for entrance to the Indian service, and the non-possession of which ensures rejection. It is, in the opinion of the writer, not less important that a standard of hearing power should be formed for the same purpose. It is indeed probably of greater importance that a fuller examination be made of the aural organ and its acuteness, than of the visual organ and its function. For, as the loss of function by an abnormal refraction—which is the great cause of defect—can be remedied by the use of lenses, and make the candidate as fit, with those lenses, for the service as a candidate with normal refraction, so may the remedy of altered tension and position of the aural conducting apparatus be applied during the medical examination so as to give a normal acuteness of function. But this full perception may not be present a few hours afterwards, and may be concurrent with tissue changes of a kind which should preclude the acceptance of the candidate. The spectacles are apparent to all, and will, as a rule, remedy the defective refraction at all times; but the changed tension, and the means of effecting it, are discoverable only by an expert, and may be used only on the occasion of the pass-examination.

Other conditions may be present in the conducting apparatus, which, though not causing any appreciable changes in the hearing power at the time of examination, which is virtually a test in direct simple conversation only, are liable to increase, and render the candidate unfit for the service. Such are exostosis, or an old callous perforation of the membrane, which, as long as the former does not materially lessen the calibre of the meatus, or the latter effects only slight changes in position, and interferes only in a small degree with the muscular accommodation, can be determined solely by an objective examination of the parts. The exostosis may never increase to such an extent as to block the canal, but a perforation will certainly, at some time or other, lead either to acute mischief, or to an early loss of acuteness for compound sounds.

Unless the examination be conducted in a manner which will ensure the detection of loss of acuteness of function with or without mechanical aids, of irregularities which may continuously or only temporarily interfere with the faculty, and of conditions which predispose to aural disease, it will fail in its intention.

That a set of rules should be drawn up regarding the defects of hearing which disqualify candidates for admission into the services, is the purpose of this paper, so that when the private medical adviser is consulted by the guardians of a proposed candidate, he may be able to inform them as to his possessing the required acuteness of vision and hearing.

That such advice should be sought by all guardians of such candidates before entering them on a lengthy and expensive training, is the warning of the writer.—*Medical Times.*

